# Aspirin delays mesothelioma growth by inhibiting HMGB1-mediated tumor progression

**DOI:** 10.1038/cddis.2015.153

**Published:** 2015-06-11

**Authors:** H Yang, L Pellegrini, A Napolitano, C Giorgi, S Jube, A Preti, C J Jennings, F De Marchis, E G Flores, D Larson, I Pagano, M Tanji, A Powers, S Kanodia, G Gaudino, S Pastorino, H I Pass, P Pinton, M E Bianchi, M Carbone

**Affiliations:** 1 University of Hawaii Cancer Center, University of Hawaii, Honolulu, HI 96813, USA; 2 Department of Molecular Biosciences and Bioengineering, University of Hawaii, Honolulu, HI 96813, USA; 3 Department of Morphology, Surgery and Experimental Medicine, University of Ferrara, Ferrara 44121, Italy; 4 San Raffaele University and Scientific Institute, Milan 20132, Italy; 5 Samuel Oschin Comprehensive Cancer Institute and Department of Biomedical Sciences, Cedars-Sinai Medical Center, Los Angeles, CA 90048, USA; 6 Department of Cardiothoracic Surgery, New York Langone Medical Center, New York, NY 10016, USA

## Abstract

High-mobility group box 1 (HMGB1) is an inflammatory molecule that has a critical role in the initiation and progression of malignant mesothelioma (MM). Aspirin (acetylsalicylic acid, ASA) is the most widely used nonsteroidal anti-inflammatory drug that reduces the incidence, metastatic potential and mortality of many inflammation-induced cancers. We hypothesized that ASA may exert anticancer properties in MM by abrogating the carcinogenic effects of HMGB1. Using HMGB1-secreting and -non-secreting human MM cell lines, we determined whether aspirin inhibited the hallmarks of HMGB1-induced MM cell growth *in vitro* and *in vivo*. Our data demonstrated that ASA and its metabolite, salicylic acid (SA), inhibit motility, migration, invasion and anchorage-independent colony formation of MM cells via a novel HMGB1-mediated mechanism. ASA/SA, at serum concentrations comparable to those achieved in humans taking therapeutic doses of aspirin, and BoxA, a specific inhibitor of HMGB1, markedly reduced MM growth in xenograft mice and significantly improved survival of treated animals. The effects of ASA and BoxA were cyclooxygenase-2 independent and were not additive, consistent with both acting via inhibition of HMGB1 activity. Our findings provide a rationale for the well documented, yet poorly understood antitumorigenic activity of aspirin, which we show proceeds via HMGB1 inhibition. Moreover, the use of BoxA appears to allow a more efficient HMGB1 targeting while eluding the known gastrointestinal side effects of ASA. Our findings are directly relevant to MM. Given the emerging importance of HMGB1 and its tumor-promoting functions in many cancer types, and of aspirin in cancer prevention and therapy, our investigation is poised to provide broadly applicable information.

Malignant mesothelioma (MM) is an aggressive cancer that arises from the neoplastic transformation of mesothelial cells lining the pleural, peritoneal, and pericardial cavities.^1^ In the United States, ~3200 individuals are diagnosed annually with MM and a similar number dies of this almost invariably fatal disease.^1^ Major risk factors for MM are exposure to asbestos and erionite mineral fibers and germline *BAP1* mutations.^1, 2^

We have demonstrated that high-mobility group box 1 (HMGB1), the prototypical damage-associated molecular pattern molecule that is normally present in the nucleus of cells, is a critical mediator of asbestos-induced MM (reviewed in Carbone and Yang^3^). Within the nucleus, HMGB1 is a non-histone chromatin-binding protein that regulates nucleosome assembly and chromatin structure.^4^ HMGB1 is passively released by necrotic cells or actively secreted by immune and cancer cells.^4, 5, 6, 7^ When in the extracellular space, HMGB1 is responsible for the initiation and perpetuation of the inflammatory response and also directly promotes MM growth.^4, 5, 7^

Our published data show that asbestos causes necrosis of primary human mesothelial cells that results in the passive release of HMGB1 into the extracellular space where it induces the secretion of TNF-*α* and other cytokines, recruits macrophages and thus initiates inflammation.^5^ The prolonged biopersistence of asbestos fibers lodged in the pleura initiates a vicious cycle of chronic cell death and chronic inflammation that, over a period of many years, can lead to MM.^5^ As MM arises in an HMGB1-rich environment, most of the MM cells are HMGB1 growth dependent and require HMGB1 to migrate and invade nearby tissues.^7^ Accordingly, out of seven MM cell lines available in our lab, six actively secrete high amounts of HMGB1 and are dependent on it for growth.^7^ Moreover, we have demonstrated that the tumor phenotype of HMGB1-secreting human MM cells requires HMGB1 for continued growth, and that abrogation of HMGB1 function may have therapeutic efficacy.^7^

Although MM is the most well-characterized HMGB1-related tumor model,^7^ the relevance of extracellular HMGB1 to carcinogenesis has also been proposed in other inflammation-related malignancies.^8^ Recent data indicate that HMGB1 initiates a chain of events that promotes tumor metastasis in melanoma,^9^ a malignancy that shares some common molecular pathogenetic mechanisms with MM.^2^ HMGB1 levels in blood are elevated in MM,^7, 10^ and in several other inflammation-related cancers.^11, 12^

Acetylsalicylic acid (ASA), commonly known as aspirin, is the most widely used nonsteroidal anti-inflammatory drug (NSAID) worldwide.^13^ In addition to its well-known effects in reducing inflammation, and preventing platelet aggregation and related effects on cardiovascular diseases, ASA reduces the incidence, metastatic potential and mortality of colon cancer, and possibly of other solid malignancies, most of which have inflammation-mediated initiation or progression.^14, 15^

ASA is absorbed by the stomach and upper intestine and is deacetylated to form salicylic acid (SA) in about 15–30 min. SA has a half-life of several hours in human plasma; thus, much of ASA bioactivity is attributed to SA. In humans, peak levels of SA in plasma vary between 20 and 150 *μ*M at ASA doses of ≤325 mg per day and vary between 0.3 and 1.5 mM in patients taking up to 1.5 g per day of ASA.^16, 17^ These doses are hereafter collectively referred to as 'therapeutic levels' as these are the dose ranges of aspirin that are administered for different therapies to millions of people every day.

To the best of our knowledge, the possible beneficial effects of ASA in human MM have not been investigated. However, by reviewing the information collected for the Physician's Health Study,^18^ we found data suggesting a possible association between ASA use and reduced MM incidence. In this study, 22 067 physicians were followed for 24 years with 17 reported cases of MM. An intention-to-treat analysis comparing ASA (325 mg on alternate days) and placebo treatment revealed a relative risk of 0.7 (0.3–1.9, 95% confidence interval), indicating a 30% reduction in MM risk. The small number of MM cases resulted in insufficient power for a statistically significant analysis; however, these findings suggested that ASA might have the potential as an effective drug for MM. In the present study, we demonstrate that ASA and its metabolite, SA, exert anticancer activity against MM by inhibiting the biological effects of HMGB1 at physiologically relevant concentrations.

## Results

### Treatment with ASA significantly reduces MM tumor growth *in vivo*

**Figure 1:**
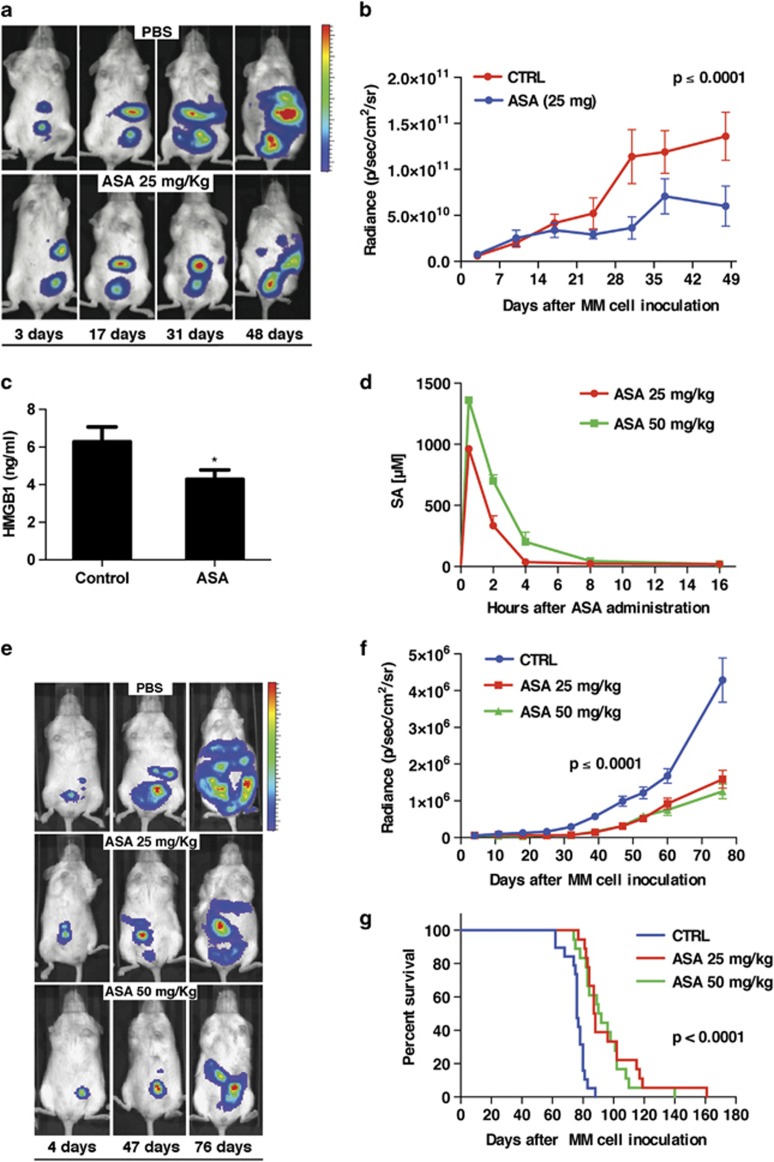
Treatment with ASA significantly reduces MM tumor growth *in vivo.* Fourteen SCID mice were injected intraperitoneally with 5 × 10^5^ REN/luc cells. After formation of detectable tumor nodules by IVIS imaging (4 days after cells injection), mice were weighed and randomly assigned to control (gavage of vehicle solution) and treatment (gavage of 25 mg/kg per day ASA); groups of seven animals each. (**a**) Representative IVIS images. (**b**) Tumor growth curves of the two groups. Based on a slope comparison test, ASA treatment resulted in significant suppression of MM growth compared with controls (*t*(67)=4.8, *P*<0.0001). (**c**) Female SCID mice were randomly assigned to receive ASA- (25 mg/kg) or vehicle-supplemented gavage feeding. Blood was collected by cheek bleeding and serum levels of HMGB1 were measured by ELISA. *P<0.05. (**d**) SA plasma levels in mice receiving ASA. Fifteen female SCID mice were randomly assigned to receive ASA (25 or 50 mg/kg) or vehicle by gavage feeding in groups of five mice. Blood was collected by cheek bleeding at different time points ranging from 30 min to 24 h after feeding, and plasma levels of SA were measured with a specific ELISA. (**e**) Representative IVIS images at different days postinjection of REN/luc cells shows reduced tumor growth in ASA-treated mice. Sixty SCID mice were injected intraperitoneally with 5 × 10^5^ REN/luc cells. After formation of tumor nodules detectable by IVIS imaging (4 days after cells injection), mice were weighed and randomly assigned to control (gavage of vehicle) and treatment (gavage of ASA at 25 or 50 mg/kg per day for the first 2 weeks and three times a week thereafter), groups of 20 animals each. (**f**) Tumor growth curves of the three animal groups up to 76 days. Measurement was halted thereafter as most mice in the control group had died. Based on a slope comparison test, treated animals showed a marked reduction in tumor growth (*t*(443)=9.9, *P*<0.0001). (**g**) Kaplan–Meier survival plot of the three animal groups. Median survival was 76 days in the control group, 87.5 days in the ASA 25 mg/kg group and 91 days in the ASA 50 mg/kg group. The increase in survival of treated animals is statistically significant for both ASA -treated groups (*χ*^2^(2)=38.0, *P*<0.0001, log-rank test)

To determine whether ASA influences MM growth *in vivo*, a xenograft model was established by injecting severe-combined immunodeficient (SCID) mice with luciferase-expressing HMGB1-secreting human MM cell line REN (REN/luc) intraperitoneally.^7^ ASA was given by oral gavage at the doses of 25 mg/kg per day, which according to previous studies should be equivalent to 80–110 mg per day in humans.^19^ ASA treatment was initiated 4 days after injecting REN/luc cells, which is when the tumors became detectable by IVIS imaging. ASA was administered daily thereafter, until the experiment was halted because of gastrointestinal (GI) bleeding (Figure 1a). A slope comparison test revealed that ASA treatment caused a significant suppression of MM growth compared with controls (*t*(67)=4.8, *P*<0.0001) (Figure 1b). Mice receiving ASA showed a significant reduction of HMGB1 in serum (Figure 1c). We ruled out the possibility that SA-HMGB1 interaction might impair measurement of HMGB1 by ELISA (Supplementary Figure S1). Therefore, the lower amount of HMGB1 in the serum of mice receiving ASA may be related to smaller tumor sizes.

The experiment was repeated with a modified protocol designed to reduce ASA side effects, such as GI bleeding. When the tumors were established and visible by IVIS (day 4 postinjection of REN/luc cells), ASA was administered by oral gavage daily for the first 2 weeks, and three times per week thereafter. Two different doses of ASA (25 and 50 mg/kg) were used, which yielded slightly different peak levels of SA in plasma, with the 50 mg/kg dose of ASA providing SA for a longer duration. Specifically, these doses achieved peak levels of SA of 1 and 1.3 mM, respectively, at 30 min post-ASA administration, which decreased to 0.4 and 0.7 mM, respectively, at 2 h post-ASA administration (Figure 1d).

Forty-eight days from MM cell injection, the tumor growth curves (Figures 1e and f) were comparable with those observed in the previous experiment (Figures 1a and b), and showed a marked reduction in tumor growth in animals receiving ASA (*t*(302)=11.2, *P*<0.0001, based on a slope comparison test). As there was no evidence of GI bleeding, the experiment could be continued past 48 days. Median survival was 76 days in the control group, 87.5 days in the ASA 25 mg/kg group and 91 days in the ASA 50 mg/kg group. The increased survival of treated animals was statistically significant (*χ*^2^(2)=38.0, *P*<0.0001, log-rank test) for both ASA- treated groups (Figure 1g). Overall, these data support the hypothesis that ASA/SA has therapeutic efficacy on established MM tumors.

### Salicylates inhibit colony formation of MM cells

As MM growth relies on HMGB1 and HMGB1 is a critical factor for MM pathogenesis,^7^ we hypothesized that the observed ASA antitumor effects on MM may be, at least in part, mediated via an HMGB1-dependent mechanism. Therefore, using a panel of three different patient-derived MM cell lines, REN, HMESO and PHI, we further analyzed whether ASA/SA could influence MM growth via targeting HMGB1 *in vitro*. All three cell lines secrete high amounts of HMGB1 and are 'addicted' to HMGB1 for growth.^7^ The PPM-MILL cell line, which secretes low to undetectable amounts of HMGB1 and that does not require HMGB1 to grow, was used as a control.^7^

*In vivo*, ASA is rapidly metabolized to SA; however, the kinetics of this conversion in cell culture have not been elucidated. Our data show that 1 mM of ASA (the highest concentration used in our *in vitro* experiments) was completely hydrolyzed to SA in 48 h, with 46% and 76% of ASA converted into SA after 8 and 24 h, respectively (Supplementary Figure S2). REN cells treated with therapeutic levels of salicylates showed no significant difference in viability compared with controls at 24 h (Supplementary Figure S3A) and 72 h (Supplementary Figure S3B), suggesting that ASA and SA are not directly cytotoxic.

**Figure 2:**
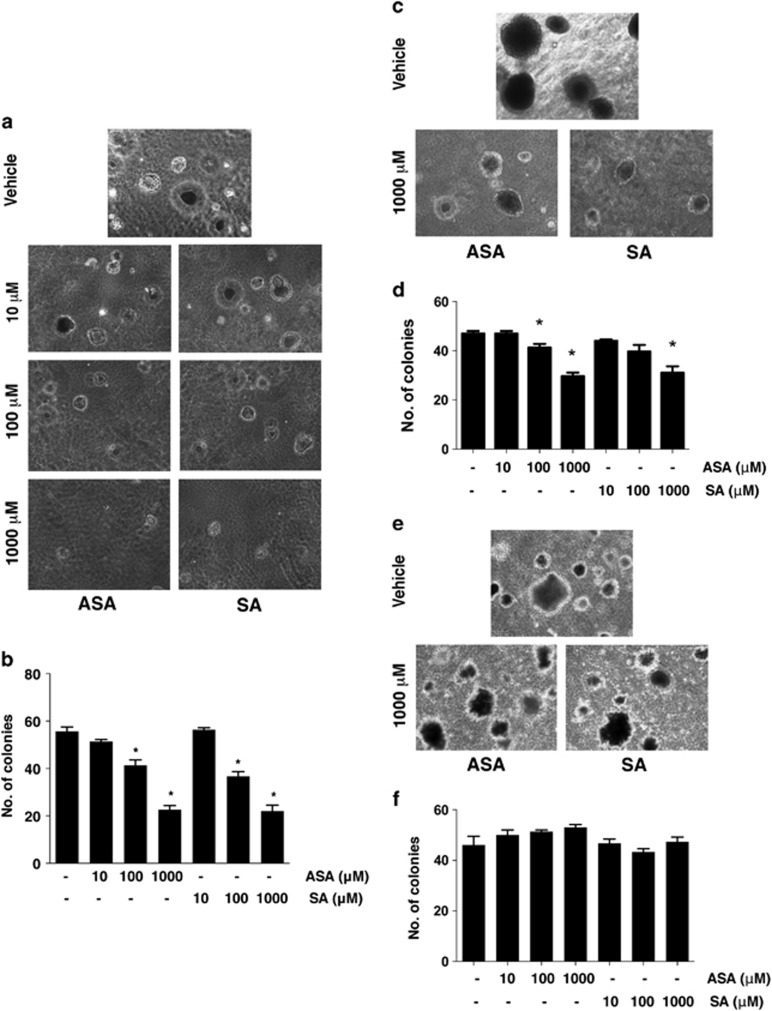
Salicylates inhibit anchorage-independent colony formation of high HMGB1-secreting REN and HMESO cells but not of low HMGB1-secreting PPM-MILL cells. Five thousand REN (**a** and **b**), HMESO (**c** and **d**) and PPM-MILL (**e** and **f**) cells were seeded on soft agar-coated plates. Fresh medium (DMEM plus 1% FBS) supplemented with different concentrations of salicylates or vehicle control was added every 2 days for 4 weeks. Representative photographs of colonies (**a**, **c** and **e**) and quantification of number of colonies (**b**, **d** and **f**). Cells were treated with vehicle or salicylates. For each well, all colonies larger than 0.1 mm in diameter were counted using the ImageJ software (NIH). In all panels, experiments were carried out in triplicate and repeated three times. *P<0.05

To determine whether anchorage-independent growth of the HMGB1-secreting MM cell lines might be suppressed by ASA/SA, MM cell lines were cultured in 6-well plates coated with soft agar for 4 weeks. Fresh medium supplemented with 1% FBS and different doses of ASA/SA or vehicle was added every 2 days. Concentrations of ASA and SA as low as 100 *μ*M significantly inhibited the formation of colonies in soft agar of high HMGB1-secreting MM cells (Figures 2a–d), whereas no effects were observed on the anchorage-independent growth of the low HMGB1-secreting MM cell line, PPM-Mill (Figures 2e and f). These findings indicate that salicylates specifically suppressed anchorage-independent growth of MM cells that secrete high amounts of HMGB1 and require HMGB1 for their growth, supporting the hypothesis that ASA exerts antitumor/MM activity through interfering with HMGB1 functions.

### Salicylates inhibit HMGB1-dependent cell motility, migration, invasion and EMT signaling

**Figure 3:**
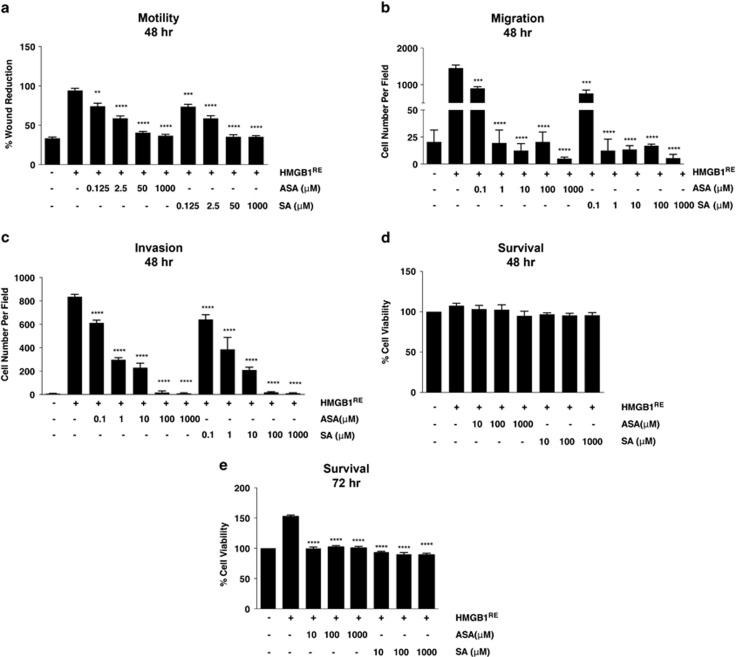
Salicylates inhibit MM cell migration, cell motility and invasion induced by HMGB1. (**a**) Quantification of wound healing assay of REN cells in the presence of HMGB1 (100 ng/ml) with or without different concentrations of ASA or SA after 48 h. The percentage of wound reduction was analyzed using the ImageJ software (NIH). Experiments were carried out in triplicate. (**b**) Both ASA and SA inhibit HMGB1-induced MM cell migration. Quantification of REN cells migrating towards HMGB1 in the presence of different concentrations of ASA or SA. Bars represent mean values per field from three fields. (**c**) Both ASA and SA inhibit HMGB1-induced MM cell invasion. Quantification of Matrigel invasion of REN cells in the presence of HMGB1 and different concentrations of ASA or SA. Bars represent mean values per field from three fields. (**d** and **e**) ASA and SA do not inhibit HMGB1-induced MM cell proliferation at 48 h (**d**), but significantly inhibit HMGB1-induced MM cell proliferation at 72 h (**e**). One thousand REN cells were treated with HMGB1 (100 ng/ml) in the presence or absence of different concentrations of ASA or SA. Cell metabolic activity was assessed by Alamar Blue assay after 48 and 72 h. Effects of treatments were assessed by analysis of variance (ANOVA) with Bonferroni-corrected posttests. (Here we are comparing MM cells treated with HMGB1 in the presence or absence of ASA/SA.) **P<0.01, ***P<0.001 and ****P<0.0001

To determine whether ASA inhibited HMGB1-dependent cell motility, migration and invasion, REN cells were treated with 100 ng/ml of reduced HMGB1, the isoform of HMGB1 that has chemoattractant activity,^7, 20^ which induced cell motility, migration and Matrigel invasion (Figures 3a–c). ASA and SA inhibited all HMGB1-induced effects at concentrations as low as 0.1 *μ*M 48 h after administration (Figures 3a–c). In contrast, HMGB1 did not induce chemotactic migration of PPM-MILL cells (Supplementary Figure S4) and ASA/SA had no influence on PPM-MILL cells in any of the assays (Figures 2e and f). To rule out the effects may be due to changes in cells viability, we examined the effect of ASA and SA on HMGB1-induced cell growth. We found that at 48 h, HMGB1-induced REN cell growth was only slightly increased and that ASA and SA did not impact cell viability (Figure 3d). Therefore, the significant inhibitory effects of ASA and SA seen at 48 h on cell migration, motility and invasion were not caused by changes in cell proliferation. However, at 72 h, HMGB1 induced significant cell growth above background, an additional mechanism by which HMGB1 promotes MM, and at this time point, ASA/SA inhibited HMGB1-induced cell growth (Figure 3e). Similar results were obtained with other HMGB1-secreting MM cells (Supplementary Figure S5).

**Figure 4:**
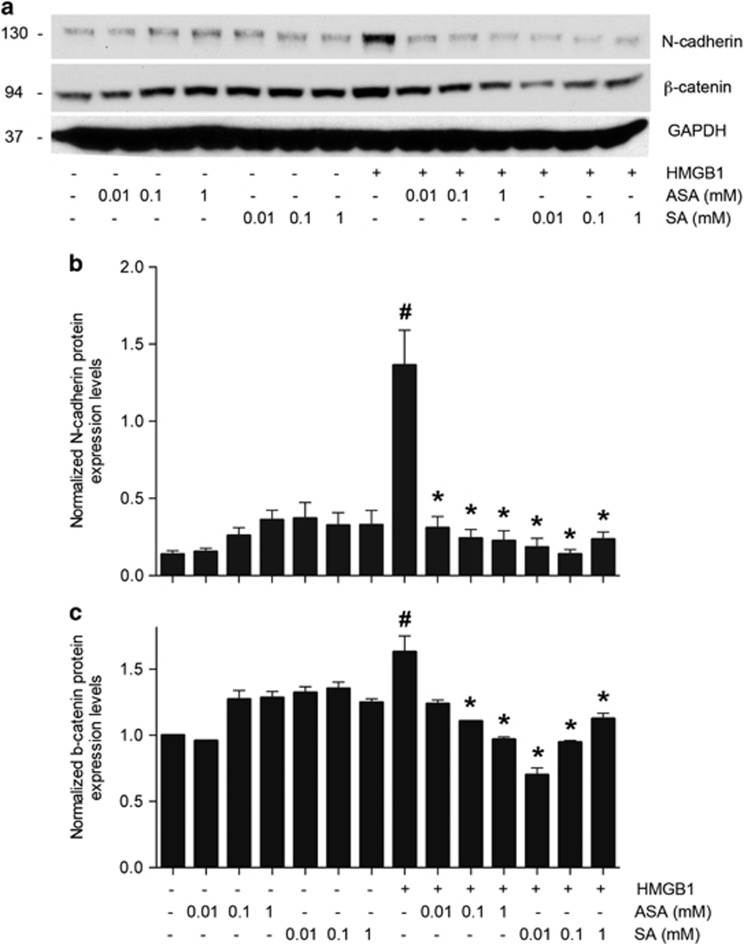
Salicylates inhibit N-cadherin and *β*-catenin expression induced by HMGB1. (**a**) Representative western blots show N-cadherin and *β*-catenin expression in REN MM cells treated with HMGB1 in the presence or absence of different concentrations of ASA or SA. (**b** and **c**) The expression of N-cadherin (**b**) and *β*-catenin (**c**) relative to glyceraldehyde 3-phosphate dehydrogenase (GAPDH) levels was evaluated as band density using the ImageJ software (NIH). ^#^Significantly different compared with MM cells not treated with HMGB1. *Significantly different compared with MM cells treated with HMGB1 in the absence of salicylates (*P*<0.05)

To investigate whether ASA and SA influences epithelial–mesenchymal transition (EMT) in MM cells induced by HMGB1, we analyzed the expression of N-cadherin and *β*-catenin in REN and PHI cells, upon treatment with ASA and SA in the presence of HMGB1 (Figure 4 and Supplementary Figure S6). As shown in Figure 4, we observed that HMGB1 increased significantly the levels of both N-cadherin and *β*-catenin and these effects were inhibited by ASA/SA, indicating that ASA and SA inhibited the expression of adhesion molecules and EMT signaling induced by HMGB1. We also found that HMGB1 induced p-AKT and that this activity was significantly reduced in the presence of ASA (Supplementary Figure S7). Inhibition of AKT activity may contribute to the overall inhibitory effect of ASA/SA on HMGB1-induced cell growth.

Overall, these results, together with the data from the colony formation assay (Figure 2), support the hypothesis that ASA/SA specifically act on high HMGB1-secreting MM cells. These data demonstrate that at concentrations ranging from 0.1 *μ*M to 1 mM, which are the concentrations obtained in human serum, following ingestion of therapeutic levels of aspirin, salicylates effectively suppress the chemoattractant activities of HMGB1 in HMGB1-secreting MM cells and suppress the MM malignant phenotype induced by HMGB1.

### Effect of salicylates on HMGB1-induced cell migration is independent of COX-2 and is not additive with the HMGB1 antagonist BoxA

**Figure 5:**
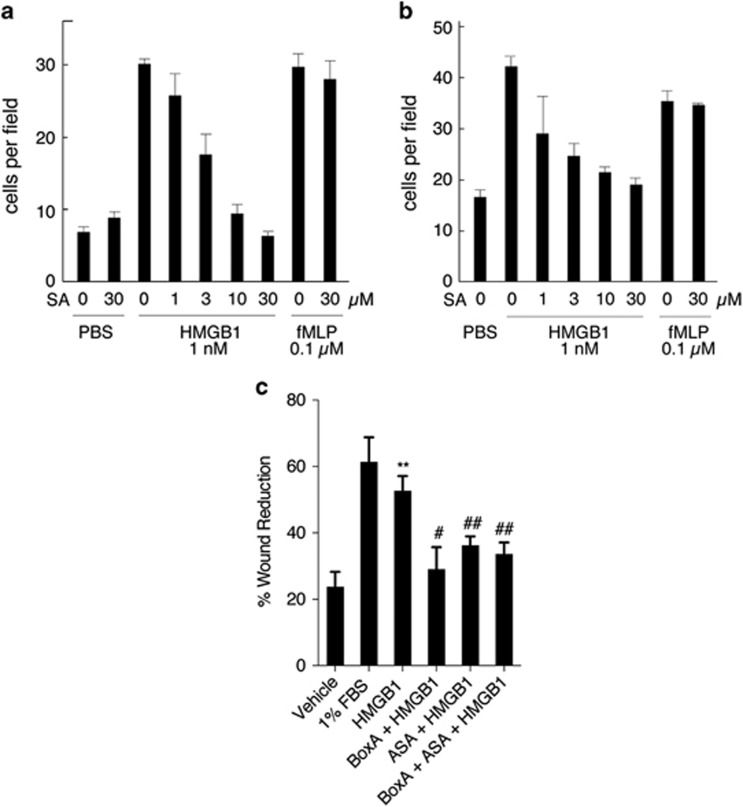
Effect of salicylates on HMGB1-induced cell migration is COX-2 independent and is not additive with BoxA. (**a** and **b**) SA suppresses HMGB1's chemoattractant activity on mouse 3T3 fibroblasts (**a**) and MEFs knock out for COX-2 (*Ptgs2*−/−) (**b**). Cell migration was performed in Boyden chambers 3 h after stimulation with 1 nM reduced HMGB1, 0.1 *μ*M fMLP or buffer (phosphate-buffered saline (PBS)). Bars represent the mean of triplicate samples (*P*<0.001 in analysis of variance (ANOVA)). (**c**) Quantification of wound healing assay of PHI cells in the presence of HMGB1 and ASA or BoxA alone or in combination after 48 h. The percentage of wound reduction was analyzed using the ImageJ software (NIH). Experiments were carried out in triplicate. **P<0.002, HMGB1-treated cells compared with vehicle. ^#^*P*=0.01 and ^##^*P*<0.005, MM cells treated with HMGB1 in the presence of BoxA, ASA or BoxA+ASA compared with cells treated with HMGB1 alone

Extracellular reduced HMGB1 acts as a chemoattractant and mediates migration of different cell types, including mouse embryonic fibroblasts, mononuclear cells and ovarian cancer cells.^21, 22, 23^ The primary action of ASA in mammals has been attributed to its effects on cyclooxygenase enzymes (COX),^13, 14^ and COX-2 deficiency impairs cell adhesion and migration of macrophages.^24^ To test whether SA-mediated inhibition of chemotaxis was mediated via COX-2 rather than HMGB1, we used mouse embryonic fibroblasts (MEFs) with ablation of the gene coding for COX-2 (*Pgts2*)^25^ and performed the cell migration assay using both *Ptgs2*−/− MEFs and wild-type (WT) fibroblasts. Our data show that SA effectively inhibited HMGB1-induced fibroblasts migration in a dose-dependent manner, in both *Ptgs2*−/− MEFs and WT fibroblasts, with an IC50 of ~3–4 *μ*M (Figures 5a and b). In contrast, SA did not affect cell migration induced by the chemoattractant *N*-formyl-methionyl-leucyl-phenylalanine (fMLP) (Figures 5a and b). Thus, SA appears to inhibit specifically the chemoattractant activity of HMGB1, rather than suppress cell migration *per se*. To further investigate whether the antichemoattractant effects of salicylates on MM cells are through COX inhibition or not, we compared the effects of ASA to indomethacin, a nonselective inhibitor of COX, and we found that ASA, but not indomethacin, inhibited HMGB1-induced motility of MM cells (Supplementary Figure S8). In addition, we found that ASA and BoxA, a specific HMGB1 antagonist,^26^ did not have synergic or additive effects, as it would have been expected if they acted through different pathways (Figure 5c). Taken together, these data support the hypothesis that the antichemoattractant effect of ASA is largely mediated via HMGB1-dependent mechanisms and it is COX-2 independent.

### Specific inhibition of HMGB1 significantly reduces tumor growth *in vivo*

**Figure 6:**
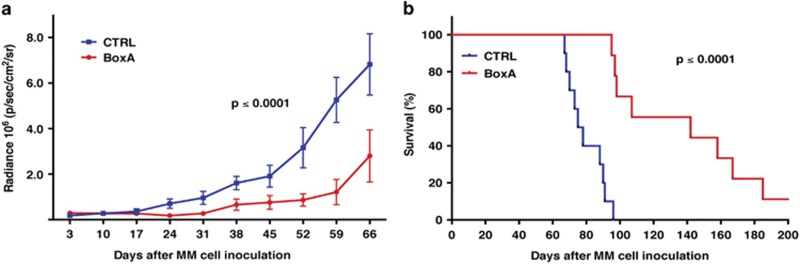
BoxA significantly reduces tumor growth in an MM xenograft model. (**a**) Tumor growth curves of BoxA-treated mice and controls. Based on a slope comparison test, tumors in BoxA-treated mice grew significantly slower than controls (*t*(146)=4.8, *P*<0.0001). (**b**) Survival curve of the two groups. BoxA-treated mice had a median survival of 142 days, compared with 76.5 days median survival in the control group in which the longest survival was 96 days. Based on the log-rank test, this difference was statistically significant (*χ*^2^(1)=18.4, *P*<0.0001)

Following the identification of a common pathway of action of ASA/SA and BoxA, we tested the efficacy of BoxA *in vivo*. Twenty SCID mice were injected intraperitoneally with 5 × 10^5^ REN/luc cells. After formation of tumor nodules detectable by IVIS imaging (4 days after injection of REN/luc cells), mice were weighed and randomly assigned to control (PBS) and treatment (400 *μ*g BoxA injection) groups of 10 animals each. Mice were injected intraperitoneally three times a week for a total of 10 weeks (12 mg BoxA/mouse in the treatment group). Based on a slope comparison test, tumors in BoxA-treated mice grew significantly slower than controls (Figure 6a, *t*(146)=4.8, *P*<0.0001), and these mice had a median survival of 142 days, compared with 76.5 days median survival in the control group in which the longest survival was 96 days (Figure 6b). Based on the log-rank test, this difference was statistically significant (*χ*^2^(1)=18.4, *P* <0.0001). One animal in the BoxA-treated group survived 413 days when eventually it became ill and had to be killed. Necropsy revealed that this mouse died of a CD3+ T-cell lymphoma (Supplementary Figure S9), a tumor type that is frequent in SCID mice. Pathological and immunohistochemical studies revealed no evidence of residual human MM cells in this mouse.

## Discussion

We report that salicylates inhibit the activities of extracellular HMGB1 and that at least part of the anticancer effects of aspirin are due to inhibition of HMGB1's activities and are COX-2 independent.

The primary mechanism of action of aspirin in mammals has been attributed to the disruption of eicosanoid biosynthesis through the irreversible inhibition via acetylation of COX, thereby altering the levels of prostaglandins.^13, 14^ Aspirin's protective effect against cancer has therefore been associated with the inhibition of COX. In supporting this, studies have shown that: (i) other NSAIDs that, similar to aspirin, inhibit COX activity, also reduce cancer risk, (ii) COX-2 is overexpressed in many tumors, and (iii) *Ptgs2* (*Cox-2*)-null mice have much reduced number of precancerous colon polyps.^27^ In contrast, other studies challenge the hypothesis that COX inhibition is responsible for the anticancer effects of aspirin and other NSAIDs,^28^ because: (i) inhibition of cancer cell growth by NSAIDs cannot be reversed by addition of prostaglandins, (ii) genetic suppression of *Cox-1* and/or *Cox-2* expression often does not alter sensitivity to NSAIDs, and (iii) some derivatives, metabolites or enantiomers of several NSAIDs retain anticancer activity despite having lost their ability to inhibit COX-1/2. Taken together, these studies argue that mechanism(s) independent of inhibition of the COX activity of COX-1/2 are, at least partially, responsible for the anticancer activity of aspirin and other NSAIDs, a conclusion also reached by others.^29^

In the present study, we examined whether aspirin and its metabolite, SA, exert anticancer activity against MM by inhibiting the biological effects of HMGB1 at physiologically relevant concentrations. We found that ASA significantly inhibited MM growth in a xenograft model, a result that was reproduced using gavage feeding in two independent experiments. The mean survival of mice treated with 25 or 50 mg/kg ASA were significantly higher as compared with untreated control mice (Figure 1). Therapeutic levels of ASA and SA suppressed the migration, invasion, wound healing, EMT signaling and anchorage-independent colony formation of HMGB1-secreting MM cells but not those of MM cells, which secrete low to undetectable amounts of HMGB1, suggesting that the antitumor activity of ASA/SA was related to HMGB1 (Figures 2, 3, 4). Moreover, SA inhibited HMGB1-induced cell migration in MEFs in which the gene coding for COX-2 (*Pgts2*) had been genetically ablated indicating that this effect is COX-2 independent (Figures 5a and b). These findings support the interpretation that the observed biological/antitumor effects were a direct consequence of SA-mediated inhibition of HMGB1 activities.

As aspirin, in addition to inhibiting HMGB1, has other activities, we used BoxA, a specific HMGB1 antagonist^26^ to determine whether specific inhibition of HMGB1 function mimicked the effects of aspirin. We tested whether HMGB1 inhibition alone was sufficient to account for the antichemoattractant activity and reduced tumor growth or whether other aspirin activities were also required. *In vitro*, the combination of ASA and BoxA did not increase their antichemoattractant activity (Figure 4c). The data are consistent with the two agents being in the same pathway. *In vivo*, BoxA administration produced similar antitumor effects as observed with aspirin, without causing the well-known side effects of aspirin, that is, GI bleeding. Median survival in control mice was 76.5 days and maximum survival was 96 days, whereas median survival of BoxA-treated mice was 142 days (Figures 6a and b). One of the BoxA-treated mice survived 413 days. This mouse eventually died of a mouse T-cell lymphoma and human MM cells could no longer be detected in it (Supplementary Figure S8). In summary, BoxA was more effective than ASA in suppressing MM growth and extending survival, and mice treated with BoxA did not appear to suffer any side effects.

Although BoxA is not yet available for clinical use, aspirin is. The beneficial effects of aspirin and BoxA detected in our study were observed treating animals during the early stages of tumor growth. Our experiments were not designed to address the possible beneficial effects of aspirin and BoxA on symptomatic MM with large tumor masses. Therefore, similar to what has been proposed for colon cancer,^14^ we propose that in human MM the beneficial effects of aspirin and BoxA may be better achieved during the early phases of tumor growth and/or in a preventive/early stage setting. Thus, aspirin administration to individuals at high risk of developing MM, such as those with a history of asbestos and or erionite exposure or germline BAP1 mutation carriers,^1, 2^ may prevent or delay the growth of MM, possibly increasing life expectancy and also increasing opportunities for early MM detection, which is associated with a prolonged life expectancy.^1^ In this regard, promising new MM biomarkers are being developed to increase opportunities for early MM detection in cohorts at high risk for MM.^3, 30^

Our data present a compelling argument that at least some of the so far elusive antitumor activity of aspirin is mediated through inhibition of HMGB1 activity. Future studies shall address the precise mechanism by which ASA inhibits HMGB1. In summary, we propose a novel mechanism by which aspirin may exert its antitumorigenic properties (i.e., HMGB1 inhibition) while remaining consistent with its well-described anti-inflammatory properties. Indeed, by identifying HMGB1 as a potential target of salicylates in our experimental MM system, we provide a proof-of-concept of a novel therapeutic mechanism that is responsible for aspirin's anticancer effects. Our results are of relevance to MM, and also to other inflammation-related malignancies, such as melanoma, breast, GI, prostate, and pancreatic cancers, whose growth and development appear also supported by high levels of HMGB1.^7, 8^

## Materials and Methods

### Study design

This study objective was to investigate the HMGB1-mediated antitumorigenic effects of salicylates. To this aim, we designed *in vitro* and *in vivo* assays using high and low HMGB1-secreting MM cell lines, which exhibit different HMGB1 dependence for cell growth. Sample selection for *in vitro* studies was based on availability of high and low HMGB1-secreting MM cell lines. Sample numbers and experimental replicates for each experiment are indicated in the figure legends. MM sample selection for our *in vivo* orthotopic xenograft mouse model was based on high HMGB1 secretion levels and on robust intraperitoneally grafting in SCID mice. Sample size, randomization and *in vivo* study end points are described in the ‘Animal Studies' Section in the Materials and Methods.

### Cell cultures and reagents

The MM cell lines PPM-MILL, PHI (originally denominated as HP3; Pass *et al.*^31^), and HMESO were established from surgically resected human MM specimens and characterized for their mesothelial origin by Drs. Harvey Pass and John Minna at the National Cancer Institute (NCI, Bethesda, MD, USA).^7^ The MM cell line REN was provided by Dr. Steven Albelda (University of Pennsylvania, Philadelphia, PA, USA). This line was originally derived from an explant of an epithelial mesothelioma. MM cell line characterization is performed routinely in our lab by immunostaining using specific antibodies against mesothelial markers, including WT-1, calretinin and pancytokeratin. All MM cell lines routinely tested negative for mycoplasma contamination. All cell lines were cultured in Dulbecco's modified Eagle's medium (DMEM; Gibco, Grand Island, NY, USA), supplemented with 10% FBS at 37 °C in a 5% CO_2_ atmosphere. REN/luc cells expressing luciferase were generated by our lab as described previously.^7^ MEFs were derived from *Ptgs2*−/− E16 mouse embryos: *Ptgs2*+/− mice were purchased from Taconic Biosciences (Germantown, NY, USA) and mated to obtain *Ptgs2*−/− embryos; embryos were genotyped.^25^ The 3T3 cells were purchased from ATCC (Manassas, VA, USA). Full-length, lipopolysaccharide-free purified reduced HMGB1 and BoxA were obtained from HMGBiotech (Milan, Italy). ASA, SA and indomethacin were purchased from Sigma-Aldrich (St. Louis, MO, USA) and were dissolved in ethanol. fMLP was obtained from Sigma-Aldrich. Salicylates ELISA Kit used for SA measurement was purchased from Neogen (Lansing, MI, USA). HMGB1 ELISA kit was purchased from IBL International (Hamburg, Germany).

### Cell viability assay

REN cells were plated in a 96-well plate (1 × 10^3^ per well), starved overnight and treated with HMGB1 (100 ng/ml) with or without various concentrations of ASA/SA. Cell viability was assessed using the Alamar Blue (AbD Serotec (Raleigh, NC, USA)) assay according to the manufacturer's instructions 48 and 72 h after treatment.

### Motility assay

MM cells were grown in 6-well plates to 80–90% confluence in medium supplemented with 10% FBS. The cell monolayer was then scratched with a P200 tip, serum-free medium supplemented with HMGB1 (100 ng/ml) and different concentrations of ASA or SA (ranging from 0.1 *μ*M to 1 mM) were added, and cells were allowed to close the wound for 48 h. Migration distance was photographed and measured at zero time and after 48 h using the ImageJ software (NIH, Bethesda, MD, USA).

### Migration and invasion assays

The *in vitro* cell migration and invasion assays were carried out using Costar Transwell-permeable polycarbonate supports (8.0 *μ*m pores) in 24-well plates (Corning Inc., New York, NY, USA). A total of 1 × 10^5^ MM cells were seeded in the insert in serum-free medium. For the invasion assays, the upper compartment of the support was coated with Matrigel (BD, San Jose, CA, USA). Experiments were performed in triplicate. MM cells were seeded on the top compartment of a Boyden chamber. The lower chamber was filled with serum-free medium supplemented with HMGB1 (100 ng/ml) and with or without different concentrations of either ASA or SA (ranging from 0.1 *μ*M to 1 mM), and cells were allowed to migrate or invade for either 3 (MEF and 3T3) or 48 h (MM cell lines). The invaded/migrated cells were quantified per field of view using the ImageJ software (NIH) and statistically analyzed.

### Western blotting

Total cell protein extracts were prepared using lysis buffer (Sigma-Aldrich). Fifty micrograms of protein were used for each sample. The proteins were separated on a 10% polyacrylamide gel and transferred to polyvinylidene difluoride membranes (EMD Millipore, Darmstadt, Germany). The membranes were blocked in Tris-buffered saline containing 0.05% Tween-20 (TBST) and 5% bovine serum albumin at 4 °C overnight before incubation with specific primary and secondary antibodies. The antibodies used were N-cadherin, *β*-catenin (Santa Cruz Biotechnology, Dallas, TX, USA) and GAPDH (EMD Millipore). The relative density of western blot bands was evaluated by ImageJ software (NIH).

### Soft agar assay

MM cells were incubated with different concentrations of ASA or SA (ranging from 0.1 *μ*M to 1 mM) and the anchorage-independent cell proliferation was assayed as described previously.^7^ Briefly, the cells (5 × 10^3^) were mixed with an equal volume of 0.6% agar in DMEM 10% FBS and placed on top of a 6-well plate precoated with 1.2% agar in DMEM plus 10% FBS. Cells were incubated at 37 °C with 5% CO_2_ and fresh medium (DMEM plus 1% FBS) supplemented with different concentrations of ASA or SA (ranging from 0.1 *μ*M to 1 mM) was added every 2 days. After 4 weeks of culture, the number and size of the colonies formed in each treatment were evaluated. For each well, all colonies larger than 0.1 mm in diameter were counted using the ImageJ software.

### Animal studies

SCID (NOD.CB17-SCID) female mice aged 6 to 8 weeks (Jackson Laboratories, Bar Harbor, ME) were housed and handled under aseptic conditions, in accordance with the University of Hawaii's Institutional Animal Care and Use Committee (IACUC) guidelines. An orthotopic mouse model of MM was established by intraperitoneal injection of 5 × 10^5^ REN/luc cells suspended in 500 *μ*l of PBS. Cells were visualized by luminescence after d-luciferin injection (150 mg/kg) using the *In Vivo* Imaging System (IVIS; Xenogen Corp., Alameda, CA, USA), with regions of interest quantified as total photon counts by Living Image software (Xenogen Corp.). Mice were then weighed and randomly assigned to control and treatment groups. Treatments were ASA, BoxA or vehicle solutions. ASA was first dissolved in dimethyl sulfoxide, and then diluted to 2.5 mg/ml with 0.5% carboxymethylcellulose sodium salt (Sigma-Aldrich).^19^ BoxA (400 *μ*g) was diluted in 200 *μ*l of PBS. Control (vehicle) solutions were DMSO in 0.5% carboxymethylcellulose or PBS for ASA and BoxA, respectively. ASA treatments were administrated via oral gavage, whereas BoxA was administered via intraperitoneal injection. Throughout treatment, tumor dimension was measured every seventh day as average radiance (photons/s/cm^2^/sr) by IVIS. Primary end points were tumor size and survival. The animals were killed when declared 'ill' by the vivarium veterinary team (i.e., because of tumor masses >2 cm, dyspnea, ascites, lethargy, and so on). Animals were necropsied and all abdominal organs were analyzed histologically. The cause of death for all the mice used in the xenograft model was intestinal occlusion caused by tumor nodules, except for one mouse in the BoxA-treated group, which died accidently during a blood draw to measure HMGB1 serum levels. In the first ASA mouse experiment, 14 MM-xenotransplanted SCID mice were randomized into two groups of seven animals each, receiving ASA 25 mg/kg or vehicle daily via oral gavage. Mice were treated for 48 days, and during the course of the experiment, ASA-treated animals experienced weight loss and died of GI bleeding. In the second ASA mouse experiment, 60 MM-xenografted SCID mice were randomized into three groups of 20 animals each. Treatment groups received ASA 25 or 50 mg/kg, daily for the first 2 weeks, and then three times a week thereafter. During the course of the experiment, there was no difference in body weight between the control and treatment groups, and no evidence of GI bleeding. In the BoxA *in vivo* experiment, 20 MM-xenografted SCID mice were randomized into two groups (control and treatment) of 10 animals each. Mice were treated intraperitoneal with either 400 *μ*g BoxA or vehicle (PBS) three times per week for 10 weeks (12 mg total BoxA/mouse). There was no difference in body weight between the control and treatment groups during the course of the experiment.

### Immunohistochemistry

Immunohistochemistry was performed with anti-CD3 antibodies (Abcam, San Francisco, CA, USA; rabbit polyclonal). Vectastain Elite ABC Kit (rabbit IgG; Vector Labs, Burlingame, CA, USA) was used according to the manufacturer's instructions. The staining was analyzed blindly by two board-certified pathologists (AP and MC).

### Statistical analysis

Statistical analysis is described in the Result section and in the Figure legends. Unless otherwise specified, statistical differences were evaluated by unpaired Student's *t*-test and considered significant at *P*<0.05.

## Abbreviations

ASA: acetylsalicylic acid
COX: cyclooxygenase enzymes
EMT: epithelial–mesenchymal transition
fMLP: *N*-formyl-methionyl-leucyl-phenylalanine
GI: gastrointestinal
HMGB1: high-mobility group box 1
MEF: mouse embryonic fibroblast
MM: malignant mesothelioma
NSAID: nonsteroidal anti-inflammatory drug
SA: salicylic acid
SCID: severe-combined immunodeficient
TNF-*α*: tumor necrosis factor-*α*
