# Supplementary material for: Aspirin delays mesothelioma growth by inhibiting HMGB1-mediated tumor progression
Source: Cell Death Dis. 2015 Jun 11;6(6):e1786–. doi: 10.1038/cddis.2015.153 (PMC4669834; doi:10.1038/cddis.2015.153)
Supplement: Supplementary Information [file cddis2015153x1.pdf]

Aspirin Delays Mesothelioma Growth By Inhibiting HMGB1-Mediated Tumor Progression

Haining Yang[1*], Laura Pellegrini[1], Andrea Napolitano[1,2], Carlotta Giorgi[3], Sandro Jube[1], Alessandro Preti [4], Cormac J Jennings[1], Francesco de Marchis[4], Erin G Flores[1], David Larson[1], Ian Pagano[1], Mika Tanji[1], Amy Powers[1], Shreya Kanodia[5], Giovanni Gaudino[1], Sandra Pastorino[1], Harvey I Pass[6], Paolo Pinton[3], Marco Bianchi[4] and Michele Carbone[1*]

SUPPLEMENTARY INFORMATION

SUPPLEMENTARY FIGURE 1

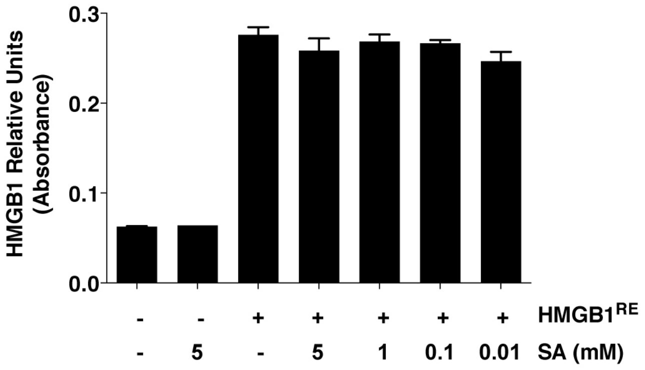

Fig. S1. SA does not interfere with HMGB1 ELISA reading. To check whether SA-HMGB1 interaction might influence HMGB1 ELISA reading, different concentrations of SA were pre-incubated for 6h at 37°C with agitation with recombinant HMGB1 (50 ng/ml). The ELISA was then performed following the manufacturer's instruction. The results demonstrate that HMGB1 ELISA reading is not influenced by SA.

SUPPLEMENTARY FIGURE 2

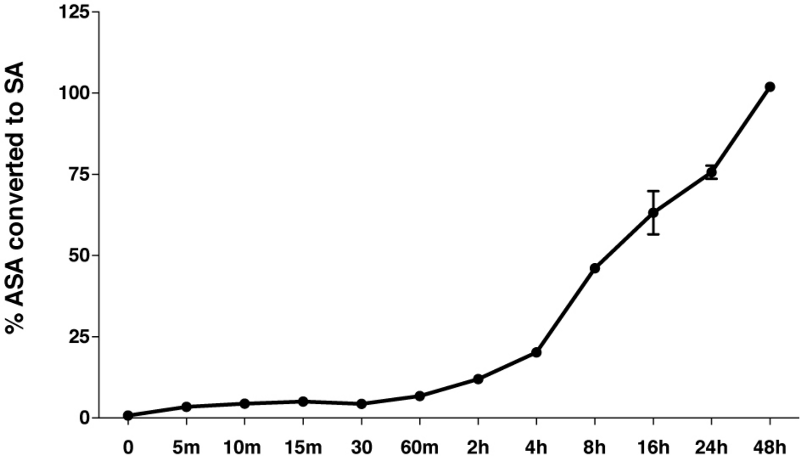

Fig. S2. ASA is converted to SA in vitro. Serum-free cell culture medium was supplemented with 1 mM ASA. Aliquots were collected at specified time points and SA levels were measured by ELISA assay. Experiments were done in duplicate and repeated three times.

SUPPLEMENTARY FIGURE 3

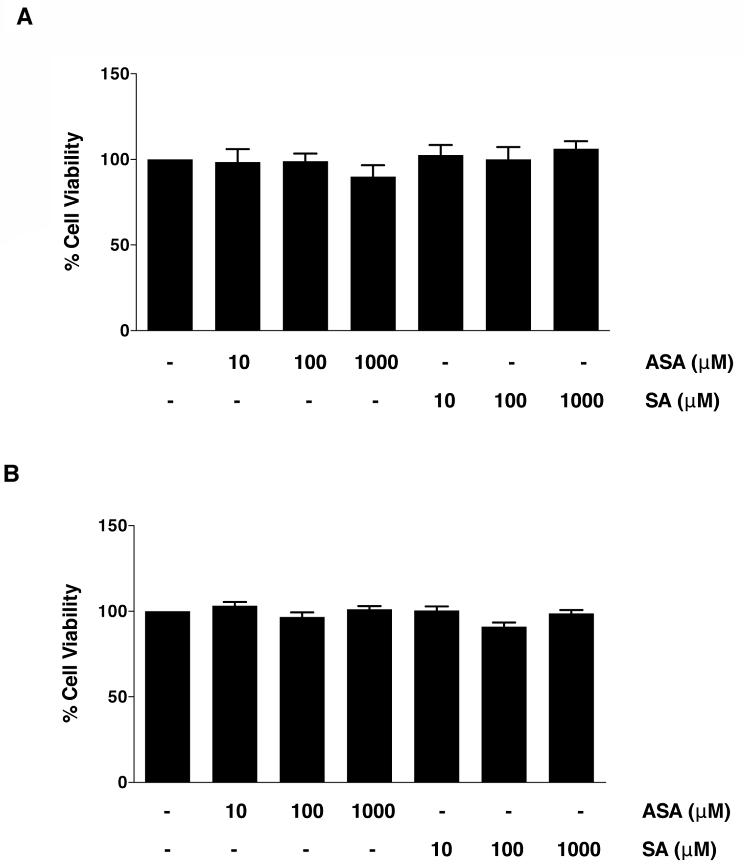

Fig. S3. Salicylates do not reduce MM cell viability. One thousand REN cells were starved overnight and then treated with different concentrations of ASA or SA. Cell metabolic activity was assessed by Alamar Blue assay after 24 hours (**A**) and 72 hours (**B**). Experiments were done in triplicate and repeated three times.

SUPPLEMENTARY FIGURE 4

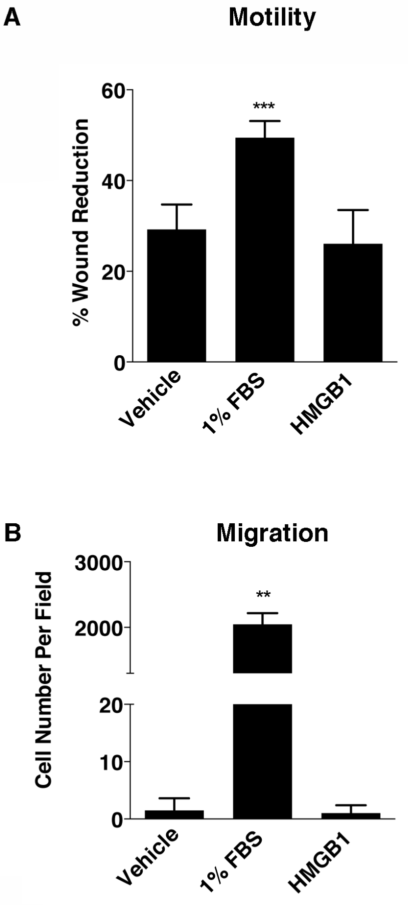

Fig S4. HMGB1 does not induce motility and migration of low HMGB1-secreting MM cell line, PPM-MILL cells. (**A**) Quantification of wound healing assay of PPM-MILL cells in the presence of either 1% FBS as positive control or HMGB1 (100 ng/ml) after 48 hours. The percentage of wound reduction was analyzed using ImageJ software. Experiments were done in triplicate. (**B**) Quantification of PPM-MILL cells migrating towards HMGB1 (100 ng/ml) or 1% FBS (positive control). Bars represent mean values per field from 3 fields. **, $p<0.01$; ***, $p<0.001$.

SUPPLEMENTARY FIGURE 5

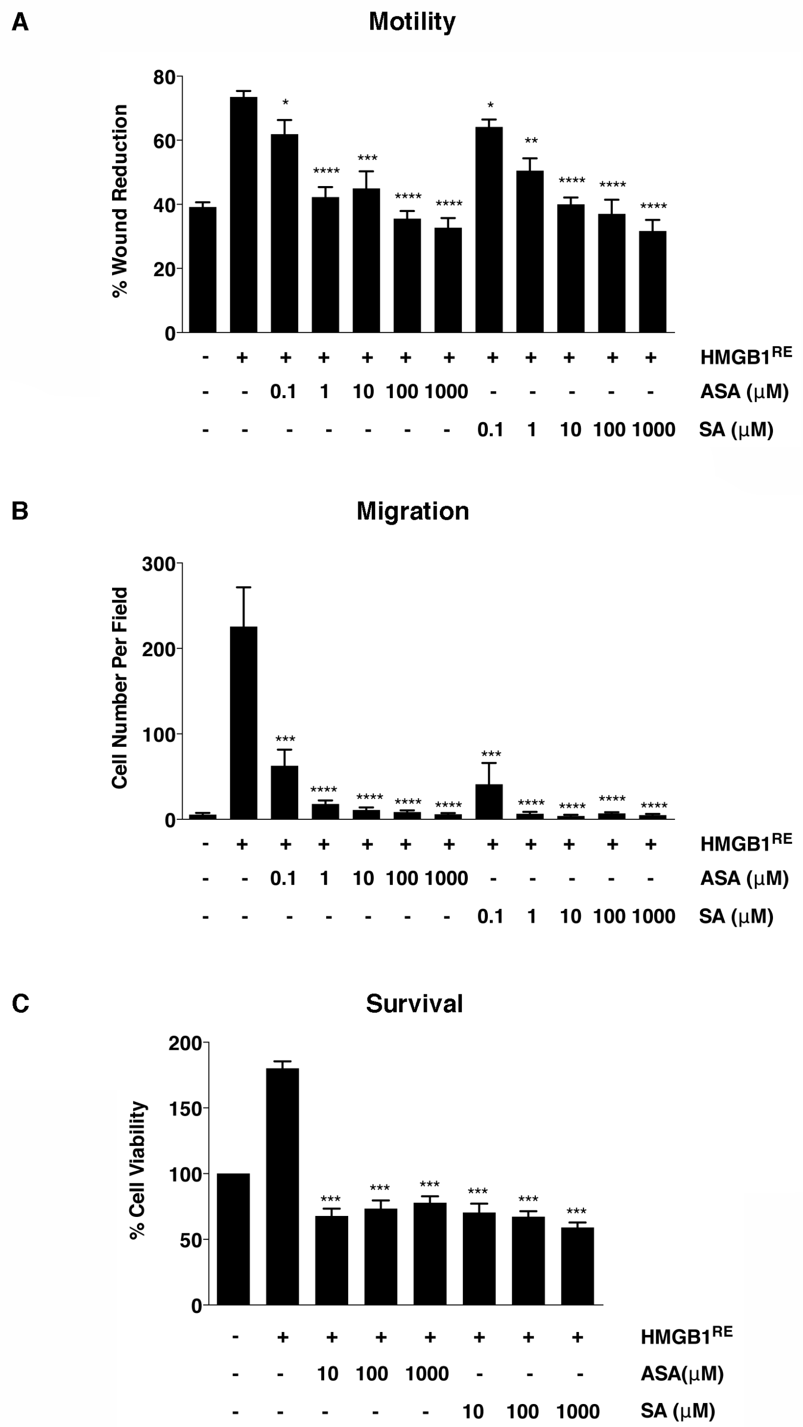

Fig. S5. Salicylates inhibit HMGB1-induced migration, cell motility in high HMGB1-secreting MM PHI cells. (**A**) Quantification of wound healing assay of PHI cells in the presence of HMGB1 (100 ng/ml) with or without different concentrations of ASA or SA after 48 hours. The percentage of wound reduction was analyzed using ImageJ software. Experiments were done in triplicate. (**B**) Quantification of PHI cells migrating towards HMGB1 (100 ng/ml) in the presence of different concentrations of ASA or SA. (**C**) Quantification of cell viability in the presence of HMGB1 (100 ng/ml) with or without different concentrations of ASA or SA after 72 hours by Alamar Blue assay. Bars represent mean values per field from 3 fields. **, $p<0.01$; . ***, $p<0.001$; . ****, $p<0.0001$.

SUPPLEMENTARY FIGURE 6

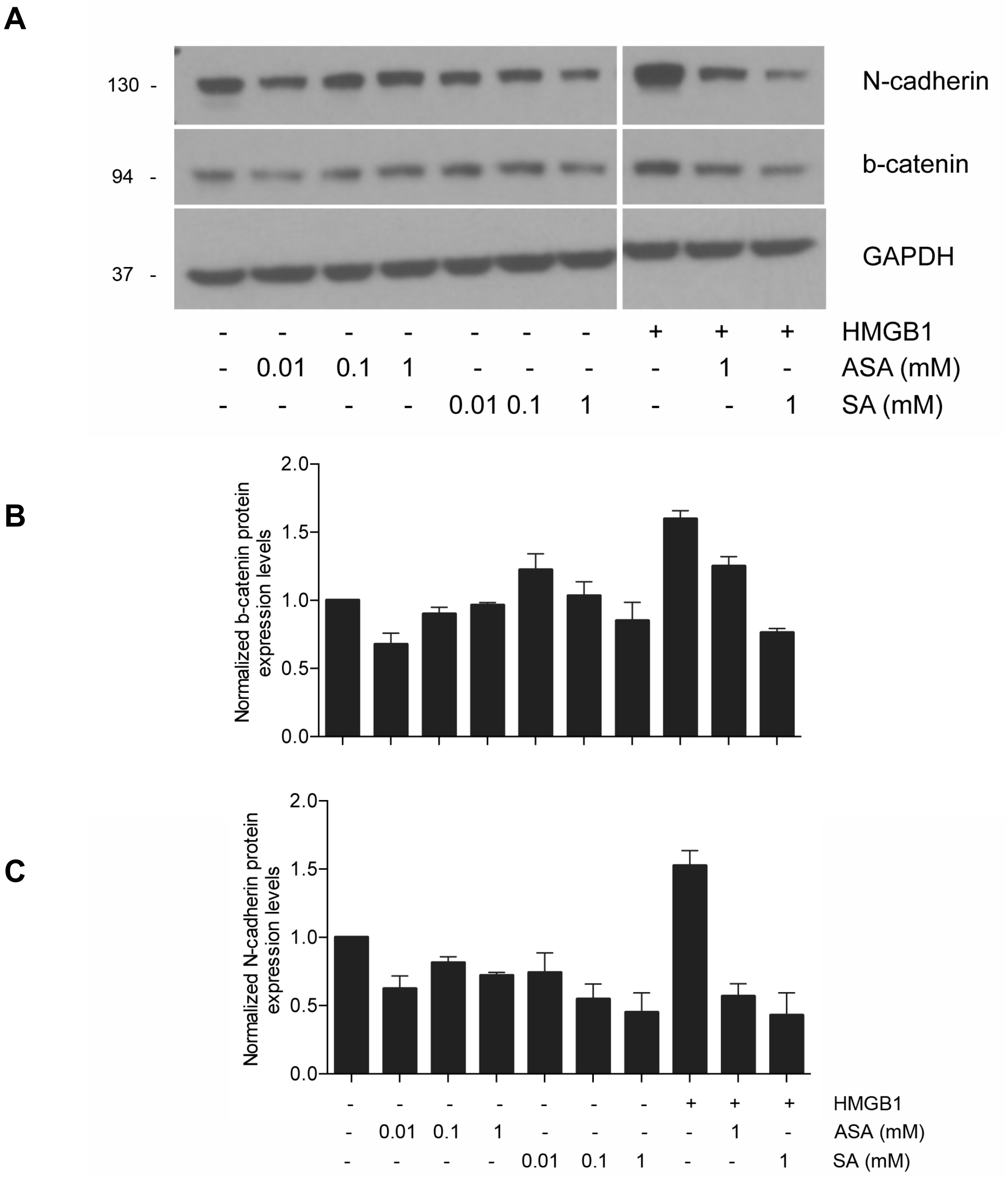

Fig. S6. ASA/SA inhibit N-cadherin and β-catenin expression induced by HMGB1. (A) Representative Western blots show N-cadherin and β-catenin expression in PHI cells treated with HMGB1 in the presence or absence of different concentrations of ASA or SA. **(B and C)** The relative expression of N-cadherin **(B)** and β-catenin **(C)** versus GAPDH was evaluated by bands relative density using ImageJ software.

SUPPLEMENTARY FIGURE 7

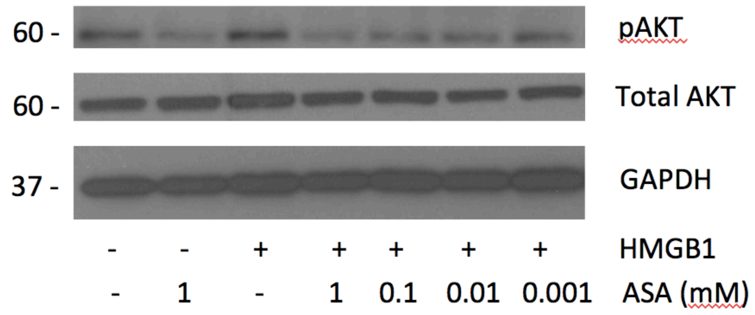

Fig. S7. ASA inhibits AKT activity induced by HMGB1. Representative Western blots show p-AKT and total AKT expression in MM cells treated with HMGB1 in the presence or absence of different concentrations of ASA.

SUPPLEMENTARY FIGURE 8

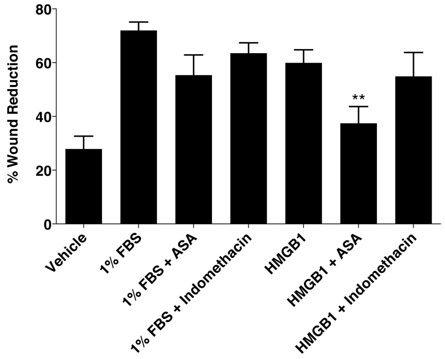

Fig. S8. Effects of different anti-inflammatory drugs on MM cells motility. Wound closure in PHI cells induced by HMGB1 after ASA (100 μM) and indomethacin (100 μM) treatments. The percentage of wound reduction was calculated using ImageJ software after 48 h of treatments. Experiments were done in triplicate. **, $p<0.01$.

SUPPLEMENTARY FIGURE 9

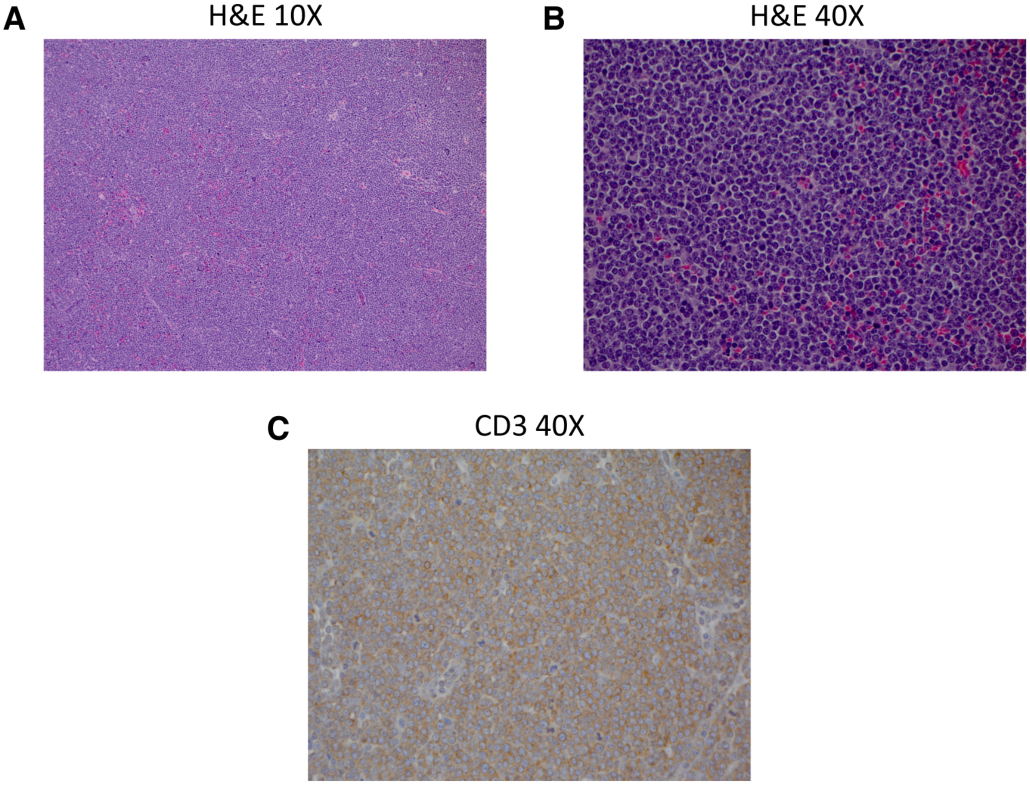

Fig. S9. T-cell lymphoma in one BoxA treated SCID mouse. Representative histological sections from the thoracic and peritoneal neoplasms. The tumors within the thorax and peritoneum were identical and consisted of a monotonous population of lymphoid cells. The cells were uniformly CD3+, suggesting a T-cell neoplasm. (**A**) H&E peritoneal tumor, 10X. (**B**) H&E peritoneal tumor, 40X. (**C**) CD3+ immunohistochemistry staining.
